# A Valuable Source of Promising Extremophiles in Microbial Plastic Degradation

**DOI:** 10.3390/polym16152109

**Published:** 2024-07-24

**Authors:** Van Hong Thi Pham, Jaisoo Kim, Soonwoong Chang

**Affiliations:** 1 Department of Environmental Energy Engineering, College of Creative Engineering, Kyonggi University, Suwon 16227, Republic of Korea; swchang@kyonggi.ac.kr; 2 Department of Life Science, College of Natural Science, Kyonggi University, Suwon 16227, Republic of Korea

**Keywords:** extremophiles, microplastic, biodegradation, plastic-degrading enzyme, plastic-degrading microorganism

## Abstract

Plastics have accumulated in open environments, such as oceans, rivers, and land, for centuries, but their effect has been of concern for only decades. Plastic pollution is a global challenge at the forefront of public awareness worldwide due to its negative effects on ecological systems, animals, human health, and national economies. Therefore, interest has increased regarding specific circular economies for the development of plastic production and the investigation of green technologies for plastic degradation after use on an appropriate timescale. Moreover, biodegradable plastics have been found to contain potential new hazards compared with conventional plastics due to the physicochemical properties of the polymers involved. Recently, plastic biodegradation was defined as microbial conversion using functional microorganisms and their enzymatic systems. This is a promising strategy for depolymerizing organic components into carbon dioxide, methane, water, new biomass, and other higher value bioproducts under both oxic and anoxic conditions. This study reviews microplastic pollution, the negative consequences of plastic use, and the current technologies used for plastic degradation and biodegradation mediated by microorganisms with their drawbacks; in particular, the important and questionable role of extremophilic multi-enzyme-producing bacteria in synergistic systems of plastic decomposition is discussed. This study emphasizes the key points for enhancing the plastic degradation process using extremophiles, such as cell hydrophobicity, amyloid protein, and other relevant factors. Bioprospecting for novel mechanisms with unknown information about the bioproducts produced during the plastic degradation process is also mentioned in this review with the significant goals of CO_2_ evolution and increasing H_2_/CH_4_ production in the future. Based on the potential factors that were analyzed, there may be new ideas for in vitro isolation techniques for unculturable/multiple-enzyme-producing bacteria and extremophiles from various polluted environments.

## 1. Introduction

Plastic materials are an inescapable part of the modern world. There are no comparable alternatives to plastic; thus, it is a challenge to ban plastic in daily life. Plastics have notable advantages, including their thermoelasticity, high resistance, stability, durability, waterproofness, light weight, and low cost [1,2]. Plastic production has been utilized on a large scale for applications such as packaging, soft drink bottles, garbage bags, and label stickers on many objects in recent decades. After significant efforts to classify plastics, less than 9% of plastic according to the United Nations Environment Program (UNEP) is recycled, 12% is incinerated, and 79% is remediated in landfills; thus, there is an enormous impact on the environment and living species at the end-of-life and disposal stage of plastics [3,4,5]. The dominant solid waste observed in the oceans is plastic, which damages the aquatic ecosystem [6]. Importantly, in the food chain, plastics have been detected at levels that are dangerous for human health [7]. Microplastics enriched with polycyclic aromatic hydrocarbons (PAHs) have been investigated to assess their probable cancer risk when ingested [8]. Methods of disposal involving incineration can limit plastic in dumps and landfills and recover heat energy. However, incineration raises serious concerns regarding its secondary pollutants (CO, NOx, dioxins, etc.), which increase air pollution and cause greenhouse effects. Although the use of catalysts has been evaluated to recover monomers and other chemicals from plastic waste, their efficiency is not stable [9]. Plastics are polymers formed by hundreds to thousands of monomers composed of chains of carbon atoms with hydrogen, oxygen, nitrogen, and sulfur atoms, leading to a low rate of degradation under natural conditions. Plastic accumulation negatively affects open environments on a global scale due to the incomplete degradation of micro- and nano-sized particles in both terrestrial and aquatic ecosystems [10,11].

Recently, numerous studies have investigated the use of typical whole-cell microorganisms that produce enzymes capable of biocatalysis during plastic degradation [12,13,14,15]. Microorganisms are considered a pool for identifying depolymerases and other key enzymes involved in plastic biodegradation. They use polymers as carbon and nitrogen sources for growth, enzyme release, and the synthesis of other valuable bioproducts. Such enzymes are classified into the hydrolase family, including cutinase, esterases, lipases, depolymerase, and PETase enzymes [16]. Polyethylene terephthalate (PET) was previously considered to be recalcitrant to biological degradation. However, some PET films, fibers, and fabrics have been reported to be degradable by PET-hydrolyzing enzymes that exhibit hydrolytic activity [17,18,19]. Additionally, to increase their flexibility, transparency, durability, and longevity, industrial plastics contain additives such as flame retardants (polychlorinated biphenyls and polychlorinated naphthalenes), plasticizers (bisphenol A and phthalate), and UV stabilizers (benzotriazoles). Therefore, well-adapted microorganisms in harsh conditions are more attractive for investigations of their roles and mechanisms that contribute to the biodegradation of plastic. They develop a remarkable diversity of mechanisms in polluted environments. However, there is a lack of knowledge and research on plastic-degrading enzymes in microbes, especially extremophiles, which were first introduced by McElroy in 1974 [20]; these are defined as organisms that thrive in physically or geochemically extreme conditions and their environmental habitats. Therefore, the present review outlines the crucial contributions, advantages, and disadvantages of the microbial degradation of plastics. Moreover, the potential applications of extremophilic bacteria are thoroughly discussed to explore their intriguing and possibly significant roles in plastic-polluted environments. It is of utmost importance to develop a new and promising green strategy for the management and treatment of (micro-) plastic waste.

## 2. Classification and Identification of Plastics

There are three general types of plastics based on their chemical structure, physiochemical properties, and biodegradation abilities.

### 2.1. Classification Based on Chemical Structures and Temperatures 

**Figure 1:**
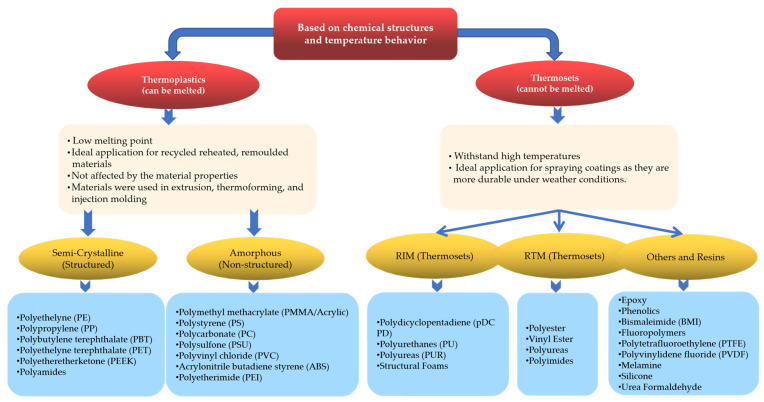
Classification of plastics based on chemical structure and temperature behavior.

Plastics are polymeric materials that are either capable of being shaped and melted or not. These materials include thermoplastics and thermosets. The general difference between these two types is that thermoplastics can be reheated, remolded, cooled, and recycled without any changes in their chemical properties. Thermosets are materials that are strengthened with heat and cannot be remolded or heated. This means that thermoplastics have low melting points, whereas thermosets can withstand higher temperatures without any changes in their original shape. Examples of each type of plastic are shown in Figure 1.

The use of thermoplastics and thermosets has several advantages and disadvantages, as listed in Table 1. 

Thermoplastics are divided into two key categories: semicrystalline and amorphous. Amorphous materials are normal solids without a specific molecular structure and with low shrinkage (PE); they tend to have lower chemical resistance and high friction. Crystalline thermoplastics have a crystalline structure with molecular chains that are packed together and systematized. These plastics are typically tougher, less fragile, and have a higher heat-distortion temperature. The use and application of both methods have advantages and disadvantages, as shown in Table 2.

### 2.2. Classification Based on Physiochemical Properties and Biodegradability

**Figure 2:**
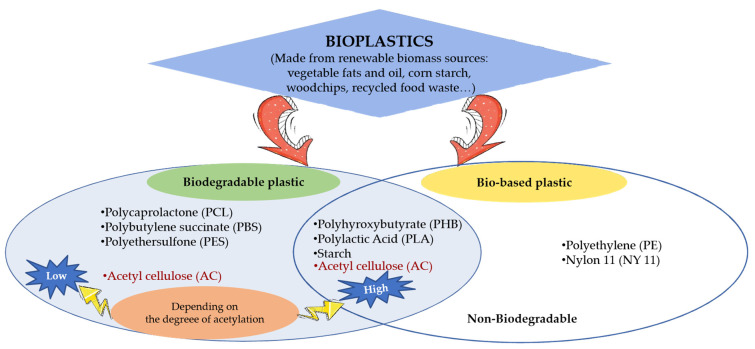
Classification of bioplastics based on properties and biodegradability.

Plastics are divided into biodegradable and non-biodegradable types. Biodegradable plastics are materials that are easily degraded by bacteria, fungi, algae, and other living organisms, as well as by abiotic elements, such as UV, oxygen, and temperature. Plastics can be broken down from complex substances into simpler organic matter. Non-biodegradable plastics cannot be decomposed by natural factors through biological processes. Thus, they remain for long periods of time and form microplastics after their disintegration and accumulation in the environment. One popular substitute that is replacing traditional plastics is known as “bioplastic”. The relationship between biodegradable and bio-based plastics is illustrated in Figure 2. 

However, the definition of “bioplastic” can be confusing. Bioplastics are biodegradable, while bio-based plastics are derived from biomass, such as plants, trees, and algae. Many types of biomass-based plastics, such as PE, PPM, PA, and PET, are not biodegradable and pose the same threat as traditional plastics [21]. On the other hand, bio-based compostable plastics, such as PLA, require specific conditions of temperature and pressure that are generally met only in industrial composting facilities [22]. Biodegradable materials can be degraded and converted into natural substances, such as H_2_O, CO_2_, and compost, using various kinds of organisms.

## 3. Biodegradation of Plastics Using Capable Bacteria

### 3.1. Investigations of Plastic-Degrading Bacteria and Their Degradation Mechanisms

**Figure 3:**
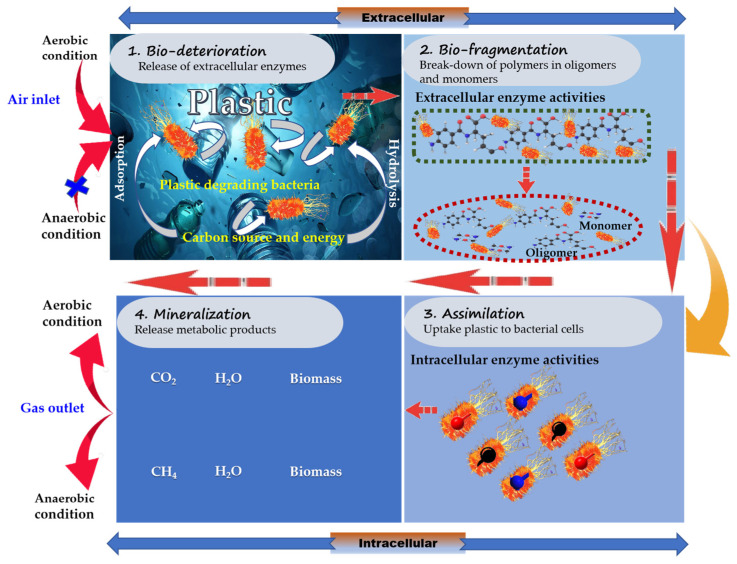
Bacterial plastic-degradation mechanisms.

The biotic components in plastics can be used by various microbial communities as metabolic intermediates and energy sources for their growth. Large polymer molecules are primarily degraded by microorganisms via extracellular processes. The released intermediates are then continuously degraded by secondary degraders. The end products include CO_2_, H_2_O, CH_4_, and new biomass with high-value bioproducts [23,24]. The general microbial degradation process of plastics is illustrated in Figure 3. Biodegradation is carried out in four main steps: (1) bio-deterioration (altering the chemical and physical properties of the polymer); (2) bio-fragmentation (breakdown of the polymer into a simpler form); (3) assimilation (microorganisms uptake polymer molecules into the cell); and (4) mineralization (oxidized metabolites are produced after the degradation process).

Several typical bacteria capable of degrading microplastics belonging to the *Bacillus*, *Brevibacillus*, *Stenotrophomonas*, *Paenibacillus*, *Pseudomonas*, and *Streptomyces* genera have been explored in previous studies [25,26,27,28]. Bacteria that produce extracellular hydrolytic enzymes, including CMCase, lipase, chitinase, protease, xylanase, and keratinase, play a crucial role in plastic degradation [15,29,30]. In another study, the PET-degrading bacterial strain *Ideonella sakaiensis* was investigated; this strain is capable of using PET as a major carbon source at mesophilic temperatures [31]. Two key enzymes responsible for the hydrolysis of PET produced by *I. sakaiensis* were identified in subsequent studies [32]. PET polymers are taken up by the whole cell and subsequently hydrolyzed by the enzyme intracellular MHET hydrolase [33]. The genes that produce polyester hydrolase, a PET hydrolytic enzyme from *Pseudomonas aestusnigri*, have also been investigated [34]. Instead of a single bacterial species, a consortium of *Enterobacter* and *Pantoea* spp. demonstrated stronger degradation efficiency on low-density PE (LDPE) [35]. In further research, a microbial consortium with thermophilic strains, such as *Brevibacillus* and *Aneurinibacillus* spp., was investigated for its high rate of polymer degradation [36]. 

However, poly-(ether urethane) polymers with benzene rings are more resistant to microorganisms than PU is, which impedes enzyme production by microorganisms, and the exploration of PU-degrading bacteria is still limited. Previous studies have investigated numerous bacterial strains that are able to deface PU, and they are dominated by the *Bacillus* and *Pseudomonas* genera isolated from various environments. Other candidates, including *Micrococcus*, *Arthrobacter*, *Corynebacterium*, and *Alternaria* spp., have also been isolated from PU-contaminated soil [37].

The decomposition of compostable plastics is carried out by mesophilic and thermophilic bacteria that can be optimized at high temperatures under aerobic conditions around 58 °C with 50% moisture [38,39]. Some studies have mentioned the anaerobic and aerobic digestion of biodegradable plastics [40]. However, the production of non-biodegradable biopolymers and the utilization of waste after use causing secondary pollution have not been discussed [41]. In another interesting investigation of anaerobic bacterial groups, plastic degradation performance was observed with a high density of methanotrophs identified with the PCR-DGGE and FISH techniques [42]. However, the efficiency of the aerobic process for plastic degradation has been reported to be higher than that under anaerobic conditions. This has led to a modern biotechnological approach in which bacteria have been more extensively investigated to address the exponential growth of plastics. The potential degradation capabilities of some bacteria are highlighted in Table 3, along with the diversity of bacteria isolated from various environmental sources.

### 3.2. Classification of Plastic-Degrading Bacterial Groups Based on Biodegradability

Plastic degradation can be classified into two key categories—biological and non-biological processes—based on the biodegradability of plastic [73]. Biodegradation is accompanied by the enzymatic and metabolic activities of microorganisms to create the end-products of carbon, water, inorganic compounds, and biomass [74]. Biodegraded polymers are broken down by microbial functional enzymes, such as hydrolases, lipases, proteases, cellulases, cutinases, and glycosidases. Generally, enzymatic degradation involves two important processes that can be measured by using weight loss and the addition of functional groups. Previous studies have demonstrated the critical role of proteases, lipases, and cutinase as special biological catalysts in the biodegradation of polymers [15,75]. Typical enzymes are classified into two main families based on their mechanisms: hydrolases and oxidase (Table 4). Previous studies have illustrated that lignin polymer-degrading enzymes, such as manganese peroxidase (MnP), lignin peroxidases, and laccases, significantly contribute to the biodegradation of PE [76]. 

## 4. Factors Affecting Plastic Degradation Efficiency

**Figure 4:**
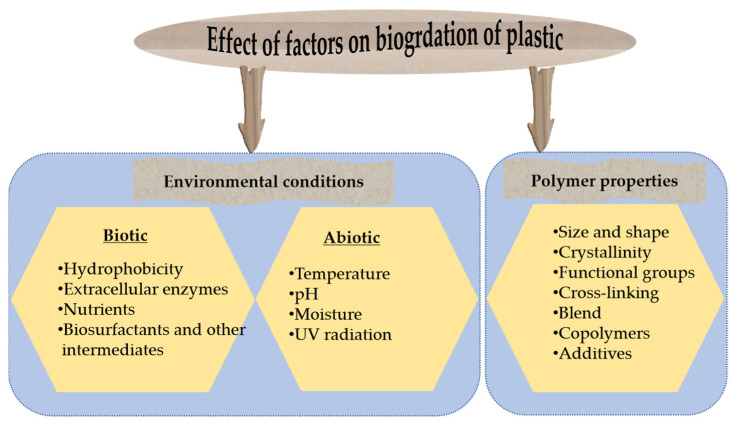
Major factors affecting plastic degradation.

Biodegradation is carried out by microorganisms through metabolic or enzymatic activities to transform the original polymers into other chemicals in an environment. Numerous factors are responsible for the biodegradation of plastics, which is generally based on the physiochemical properties of polymers and environmental conditions (Figure 4).

The degree of polymer degradation depends on the polymeric shape, size, molecular weight, functional groups on the surface, crystallinity, hydrophobicity/hydrophilicity, and additive properties. Plastic degradation is a complex process involving abiotic (non-living) and biotic (involving living) factors that increase the accessible surfaces for biodegradation [88]. The biotic degradation of plastic includes microbial action, enzymatic activity, and biodegradable ability under specific environmental conditions. Enzymes are responsible for further breaking down the fragments of polymers. In another case, enzymatic PET degradation depends on the reaction temperature and enzyme structure, as the active site accesses the surface of the polymers [89,90]. The abiotic degradation of plastic often involves exposure to sunlight, which is known as photodegradation. The energy from ultraviolet (UV) radiation can break the chemical bonds in polymer chains. The effect of UV pretreatment on the biodegradability of PE was investigated using the bacterial strain Rhodococcus rhodochrous. As a result, the UV-pretreated polymer was degraded threefold more effectively than non-treated PE [91]. Plastic can also undergo abiotic degradation through thermal processes. At high temperatures, degradation is accelerated by breaking down the polymer chains into smaller pieces. In addition, both natural and artificial chemicals significantly contribute to the abiotic degradation of plastics. Chemicals, temperature, and material properties also influence each other in reactions and determine the efficiency of plastic decomposition.

The crystallinity of a polymer is another important factor that determines its degradation rate. An increase in crystallinity can make it easier to access the amorphous region of the polymer [68,92]. PE, PP, and PVC, with their similar C-C backbone chains, have been degraded by a number of microorganisms. However, pyrolysis under anaerobic conditions has shown more efficient depolymerization of these plastic types [93]. The presence or absence of functional groups, such as carbonyl, hydroxyl, or hydrolyzable groups, can enhance or inhibit the degree of degradation.

## 5. The Special Characteristics of Extremophiles Reveal Interesting Mechanisms in Plastic Degradation

### 5.1. How Do Extremophiles Make a “Plasticsphere”?

The formation of bacterial biofilm on plastic surfaces includes the adsorption, desorption, and degradation of plastic. Besides the roughness, topography, electrostatic interactions, and hydrophobicity of plastic, which affect the attachment of microbes to plastics, an additional influence on biofilm development could also be provided by the environmental conditions, such as salinity, temperature, oxygen, and light [94]. Bacteria can survive under harsh conditions and are able to quickly adapt and reduce negative pressures from new environments [95]. During the formation of biofilms, bacteria illustrate complex growth due to the increase in antibiotic resistance through gene regulation and the enhancement of their self-protective mechanisms against unfavorable factors, such as agents, drugs, pH changes, UV radiation, and polluted environments [96]. Additionally, in biofilms, bacteria are different from those in free form, as well as those that are in both single-species and mixed-species populations [97]. They form complex multi-cellular structures and differ in metabolism. Moreover, cellular hydrophobicity plays a crucial role in attachment to the surface of plastic and in biofilm formation. The hydrophobic properties of microorganisms can significantly contribute to the degradation of hydrocarbons of biodegradable polyesters [98,99,100]. Halophilic bacteria show the ability to enhance hydrophobic interactions in environments with high salt concentrations due to their destabilized protein [101]. One of the most important mechanisms that extremophiles have been forced to develop to survive is the release of outer membrane vesicles (MVs) [102]. MVs cause a significant increase in cell surface hydrophobicity and tend to enhance the formation of biofilm [103]. Amyloid proteins (fimbriae or other microbial-surface-associated structures) are attractive because they are expressed by many types of bacteria from various habitats in biofilms. The function of amyloid fibrils has been proven to be related to the enhancement of adhesion to surfaces and biofilm formation [104]. However, studies and analyses of the role of amyloid proteins in extremophiles are a “black box” that we need to investigate in the future. Scientific evidence has not provided the conclusion that durable plastics are biodegradable. However, extremophilic microbes are still being investigated as promising candidates for some kinds of durable plastics. 

### 5.2. From Which Places Should Plastic-Degrading Bacteria Be Taken for Investigation?

It has been proposed that areas polluted by plastics or other chemical compounds with similar structures are the most suitable environments for finding well-adapted microbes. For instance, environments enriched with plastic residues, plant polymers, oil, heavy metals, and other xenobiotics are excellent habitats for extremophiles. In recent studies, the gut microbiome was explored for bacteria capable of degradation, as the microorganisms in this source protect themselves against antibiotics, pathogens, and even some kinds of toxic compounds from the food chain [105]. Moreover, multi-enzyme-producing extremophiles that can produce lignin-degrading enzymes are attractive due to their ability to transform different types of plastic [106,107,108,109]. There are still more questions than answers about their plastic biodegradation abilities.

### 5.3. Typical Extremophiles Participating in Plastic Degradation

Thermophilic bacteria are considered favorable candidates and potentially advantageous for plastic degradation because of their high growth ability, secretion of numerous oxydases and hydrolases for plastic degradation, and improved substrate bioavailability, as well as the solubility of polymers. Some typical thermophilic plastic-degrading bacteria have been investigated, such as *Enterobacter* sp., which can break down LDPE [36], *Anoxybacillus rupiensis* Ir3, which acts on nylon [110], and *Clostridium thermocellum*, which degrades PET [47].

Various pH conditions affect the solubility and softening of plastics. Acidophilic plastic degraders have been extensively investigated in the past; however, they are rarely investigated today. Some bacterial strains have been explored for their potential to degrade LDPE at pH 11, such as *Bacillus krulwichiae*, *B. pseudofirmus*, *Prolinoborus fasciculus*, and one unclassified *Bacillus* sp. [111]. A consortium of *Bacillus pseudofirmus* and *Bacillus agaradhaerens* is capable of degrading LDPE at a pH of 11 in the presence of iron-oxide nanoparticles [112].

Under extreme halophilic conditions, the genus *Erythrobacter* is predominant in natural saline environments, followed by *Hyphomonas*, *Phorimidium*, *Pseudomonas*, and *Psychrobacter*; these species have the potential to degrade PE, PP, PS, and PU [113,114]. Based on metabolic pathways, the putative enzyme phenylacetaldehyde dehydrogenase, an enzyme that participates in PS degradation, was most likely affiliated with the detected *Pseudomonas*, *Arenimonas*, and *Acidovorax* spp. isolated from industrial water samples and *Erythrobacter*, *Maribacter*, and *Mycobacterium* spp. isolated from seawater samples [115].

Psychrophiles play a crucial role in the degradation of uncrosslinked poly(ethylene-butylene-adipate) (PU-A) and slightly crosslinked poly-(ε-caprolactone) (PU-B) in marine ecosystems [115,116]. The novel/unusual properties of extremozymes and metabolic products may suggest their potential for new approaches and applications in the process of the bioremediation of plastic. Such polysaccharide-hydrolyzing enzymes have high relevance for the hydrolysis of cellulose, xylan, and starch, which are common components in natural polymers. However, there is insufficient knowledge to understand the mechanism of the lifespan of plastic with its complex interactions with the microbial communities in the environment and under laboratory conditions at low temperatures. Most PCL- and PU-degrading bacteria identified in cold environments belong to the genera *Shewanella*, *Moritella*, and *Psychrobacter* [117]. Rüthi and coworkers recently investigated several strong and effective bacterial strains that were able to degrade Impranil, such as *Amycolatopsis* sp., *Rhodococcus sovatensis*, *Psychrobacter cryohalolentis*), and *Collimonas arenae* [118].

## 6. Drawbacks and Future Prospects

The high molecular weight, lack of favorable functional groups, and crystallinity of plastics, as well as additives therein, are the main challenges in their biodegradation [119]. High-molecular-weight or long-chain plastics may inhibit microbial attack, increasing the time required for the subsequent degradation period [120]. The disadvantages of synthetic plastics include their high hydrophobicity and functional groups that are stable against the oxidation and hydrolysis of these polymers. Thus, appropriate pretreatment or additives should be considered to improve the degree of biodegradation [121]. Based on the current literature and research, the use of bacteria as a valuable source for plastic degradation still requires further investigation of isolation, enzyme production, and enzymatic activity because of the high diversity of bacteria in various natural habitats with extreme environmental conditions. For natural polymer degradation, cellulase/protease/lignin peroxidase/lipase enzymes from promising bacteria should be further investigated. On the other hand, numerous bacteria are able to utilize synthetic polymers, but the composition of polymers, especially those with additives, needs to be considered before determining a methodology for each detailed experiment. Additionally, many studies have characterized the plastisphere through taxonomic analyses, but there remains a lack of knowledge surrounding the functional potential of these communities.

Many plastic-degrading enzymes have not been well studied regarding their chemical structures and properties. Therefore, the crucial investigation and identification of novel enzymes from potential microorganisms in global metagenome/proteome datasets will provide a better understanding of the metabolic pathways of plastic degradation. In particular, screening enzymes that show high degradation activity for the most durable plastics still requires great effort from microbiologists. The close relationship between the available substrates and microbial cells may accelerate metabolic reactions that lead to changes in plastic molecules, followed by the breakdown of the plastic itself. Moreover, many current studies do not provide detailed information on polymer compositions or the additives that are present in polymer composites. Thus, it is difficult to prove the metabolites of the degradation process and their original additives. It is necessary to monitor and combine the following types of analyses: (1) assessment of the changes in polymer structure; (2) examination of the mass loss of plastic; and (3) determination of the bioproducts released during the process.

The combination of advanced molecular-based technologies, such as metagenomic and omics tools, has played a significant role in discovering the biological interactions among genes, proteins, the metabolites produced, and external environmental factors. In particular, protein engineering may help in the design of modified enzymes to improve enzyme activity and enhance plastic degradation efficiency. There is a way to standardize and optimize the conditions of the degradation process for the future development of biodegradable plastic production. Therefore, future studies need to continue to address the isolation of novel bacteria and their potential active enzymes that act on dominant plastics. It is necessary to design and screen the effects of cultural conditions on the enzyme production and metabolism of extremophilic bacteria. Abiotic factors, such as pH, temperature, and humidity, and biotic factors, such as nutrients, including carbon and nitrogen sources, can also affect the growth and activity of functional bacteria during plastic biodegradation at different levels. Recent research emphasizes the strong effect of the culturing medium on the degradation of biodegradable plastic films [118]. Therefore, it is necessary to conduct research that covers all experiments in parallel to determine the optimal conditions for each situation because many discrepancies have been observed in previous studies of degradation efficiency.

Approximately 70% of the Earth is covered by marine water, 90% of which maintains a low temperature of ~5 °C; psychrophiles are still an unexplored source [121]. Marine microorganisms include multiple extremophiles that create complex metabolic pathways in diverse communities with various mechanisms of plastic degradation. Highly active enzymes should be studied to provide deeper insight into the target of realistic short-term plastic removal. The overall methodology needs to be linked to the analysis of the structures, properties, and characteristics of polymers and the functions of bacterial strains in each growth condition in parallel. Additionally, the time taken for the evolutionary adaptation of bacteria to the presence of plastic is longer than that under normal conditions, revealing the typical activity of bacteria in complex communities during the plastic degradation process.

Long-chain polymer molecules are degraded at a low rate because of their molecular weight and tensile strength in comparison with those of short-chain polymer molecules. An exploration of the strong linear correlation between plastic loss and the main components of plastic may reveal the reason for this incomplete degradation. This would then support predictions for the enzymatic mechanism of each potential enzyme for each type of plastic. Thus, investigating novel promising bacterial candidates in terms of whole cells or depolymerase enzymes is still a long-term project. Bacteria may utilize secondary depolymerization products to produce high-value compounds that improve plastic recycling [122,123]. There is no culture condition that works best for all bacterial strains. Thus, well-adapted bacterial candidates that have been identified in harsh environmental conditions could serve as a valuable source of bio-material for plastic degradation in specific seasons and areas.

## 7. Conclusions

This review summarizes the classification and characteristics of typical plastics, and it identifies the multiple functions of bacteria with complex metabolic pathways for plastic biodegradation. Plastic-degrading bacteria are considered living bioreactors, and their enzymes act as valuable biocatalysts. The development of green technologies for effective bacterial enzymes—such as through enzyme engineering—is a promising strategy that contributes significantly to the management of the release and negative impacts of plastics on the environment and human health. An increase in plastic dumping may force microorganisms to adapt to new substrates in polluted environments. In particular, it is necessary to develop new culture methods to raise novel unculturable bacteria and extremophiles from various environments in the laboratory to investigate their unknown functions, as they are considered a genetic pool in biotechnology for a variety of applications in waste treatment and management strategies. In addition, the development of new costly plastic materials with a smaller negative impact on the environment is encouraged by using renewable raw materials. Due to the longevity of plastic in the environment and the slow adaptation of natural bacteria, effort and patience in research on plastic degradation will contribute to “fixing” and “cleaning” the consequences of our actions on the planet. Therefore, improving the efficiency of plastic degradation by enhancing the key influencing factors, such as the hydrophobicity of bacterial cells and plastics, as well as the correlation of present substrates and microorganism cells in well-controlled metabolites during plastic degradation with correct and detailed information on the original plastics, should be more deeply studied in the future.

## Figures and Tables

**Table 1:** Advantages and disadvantages of thermoplastics compared with thermosets.

	**Thermoplastics**	**Thermosets**
**Advantages**	High recyclability Can be remolded and reshaped High corrosion/detergent/chemical/chip resistance Unchanging chemical nature of the polymer Electrical insulation High aesthetic finishes Can be made in high volume with high precision Produced via eco-friendly manufacturing	Capable of molding with different tolerances Variable wall thicknesses to improve structural integrity Cheaper materials Flexible product designs Great tolerance at high temperatures High corrosion and water resistance Low thermal conductivity Strong dimensional stability Cheaper setup and tools Superb electrical insulation properties Flexible choice of color and surface finishes High strength-to-weight ratio High aesthetic finishes Cost-effective
**Disadvantages**	Tend to fracture under high stress Easy to soften when reheated Easily degrade under sunlight or UV light More expensive materials Some materials have poor resistance to hydrocarbons and organic/highly polar solvents	Cannot be remolded, reshaped, or recycled Poor thermal conductivity Difficult to surface-finish

**Table 2:** The advantages and disadvantages of semi-crystalline thermoplastics and amorphous thermoplastics.

	**Semi-Crystalline Thermoplastics**	**Amorphous Thermoplastics**
**Advantages**	Form tough plastics due to their strong intermolecular forces Provide extremely high chemical resistance Good stiffness and strength and low coefficient of friction	Easy to thermoform Dimensional stability Superior impact strength High resistance to hot water/steam and chemicals and good stiffness and strength
**Disadvantages**	Difficult to thermoform Dimensional instability causes more shrinking in the direction transverse to flow Average impact resistance Difficulties in manufacturing	More sensitive to stress cracking Limited performance as bearings/wear components Lower chemical resistance and higher friction

**Table 3:** Potential plastic-degrading bacteria.

No.	Bacteria	Source of Isolation	Plastic Types	Efficiency of Degradation (%)	References
1	*Achromobacter denitrificans*	A coastal area	PE	40% in 2 months	[43]
2	*Achromobacter xylosoxidans*	Soil	PE	9.38% in 150 days	[44]
3	*Bacillus cereus strain* A5	Surface water	PE	35.72% in 16 weeks	[45]
4	*Bacillus amyloliquefaciens*	Composted plastic material	PE	3.2% in 60 days	[46]
5	*Bacillus krulwichiae*	Rock crevices	PE	9.9% in 90 days	[47]
6	Consortia of *Brevibacillus* sp. and *Aneurinibacillus* sp.	Sewage treatment plants and waste management landfills	PE	58.21% in 140 days	[36]
7	*Paenibacillus* sp.	Soil	PE	22.46% in 35 days	[48]
8	*Microbacterium paraoxydans*	A clinical sample	PE	61% in 60 days	[49]
9	*Micrococcus luteus* IRN20	Plastic dump landfill soil	PE	18.9% in 21 days	[23]
10	*Stenotrophomonas* sp.	Landfill soil	PE	8% in 100 days	[50]
11	*Achromobacter* sp.	Drilling fluid	PE	8% in 100 days	[50]
12	*Exiguobacterium* sp.	Plastic waste	PS	12.4% (*w*/*w*) in 30 days	[51]
13	*Acinetobacter* sp.	The gut microbiome marine benthic polychaetes support	PS	12.14% in 60 days	[52]
14	*Achromobacter xylosoxidans* M9	A mealworm gut-derived solution	PS	7% in 30 days	[53]
16	*Massilia* sp. FS1903	The gut of *Galleria Mellonella* (*Lepidoptera: Pyralidae*) larvae	PS	12.97% in 30 days	[54]
17	*Pseudomonas lini* JNU01	Soil	PS	1.45% in 30 days	[55]
18	*Exiguobacterium* sp.	The gut of Tenebrio molitor larvae	PS	7.4% in 60 days	[56]
19	*Bacillus paralicheniformis* G1	Sediment samples from Arabian sea	PS	34% PS in 60 days	[57]
20	*Bacillus* sp., *Pseudomonas* sp., *Staphylococcus* sp.	Mangrove environments	PS, PET	18% in 90 days	[58]
21	*Ideonella sakaiensis* sp.	A recycling plant in Japan	PET	75% in 70 days	[59]
22	*Clostridium thermocellum*	A plant compost metagenome	PET	60% in 14 days	[15]
23	*Bacillus cereus*	Soil	PET	70–55% in 180 days	[60]
24	*Bacillus pseudomycoides*	Soil	PET	>65% in 28 days	[61]
25	*Pseudomonas citronellolis and Bacillus flexus*	Soil under pine trees and compost	PVC	19% in 30 days	[62]
27	*Klebsiella* sp.	Pest larvae	PVC	7% in 10 days	[63]
28	*Bacillus* sp. and *Micrococcus* sp.	Soil	PVC	87.3% and 91.6%, respectively in 6 months	[64]
29	*Bacillus safensis* PLA1006	Landfill soil	PLA	8% in 30 days	[65]
30	*Bacillus* sp. and *Miscanthus* sp.	A heavy-metal-contaminated environment	PLA	70% in 6 months	[60]
31	*Lysinibacillus* JJY0216	A soil grove	PP	4% in 6 days	[66]
32	*Bacillus flexus*, *Pseudomonas azotoformans*, *Bacillus subtilis*	Soil	PP	22.7% in one year	[67]
33	*Bacillus cereus* and *Sporosarcina globispora*	Mangrove sediments	PP	12% and 11%, respectively in 40 days	[68]
34	*Bacillus* sp.	Municipal compost waste	PP	12% in 15 days	[69]
35	*Bacillus paramycoides*	A river	PE, PP	78.99% in 21 days	[70]
36	*Pseudomonas putida*	Farm soils	PU	92% in 4 days	[71]
37	*Serratia sp.* HY-72	The intestine of the Asian mantis	PU	23.95% in 2 weeks	[72]

**Table 4:** Plastic-degrading enzymes produced from bacterial sources.

Enzyme Family	Enzyme Group	Enzyme-Producing Bacterial Source	Type of Plastics	References
**Hydrolase**	Esterases	*Pseudomonas antarctica*	PU	[77]
	Lipases	*Pseudomonas* sp.	PU	[25]
	Lipases	*Pseudomonas* sp.	PES	[78]
	Depolymerase (extracellular enzymes)	*Paenibacillus alvei*	PHB	[79]
	Cutinase	*Saccharomonospora viridis*	PE	[31,80]
	PETase enzymes	*Ideonella sakaiensis*	PET	[32,81,82]
**Oxidase**	Laccase	*Rodococcus rubber*	PE	[83]
	PVA dehydrogenase	*Enterobacter cloacae*	PVA	[84]
	Monoxygenases	*Rhodococcus wratislaviensis*	PE	[85]
	Alkane hydroxylase	*Rhodococcus* sp.	PE	[86]
	Lignin peroxidase	*Bacillus* sp., *Streptomyces* sp.	PE	[76]
	Manganese peroxidase	*Bacillus* sp.	PE	[87]

## Data Availability

The dataset referenced herein is available from the authors upon request.
